# Supplementary material for: Comparison of the 2013 and 2019 Nationwide Surveys on the Management of Chronic Kidney Disease by General Practitioners in Japan
Source: J Clin Med. 2022 Aug 16;11(16):4779. doi: 10.3390/jcm11164779 (PMC9410076; doi:10.3390/jcm11164779)

Supplementary Figure S1

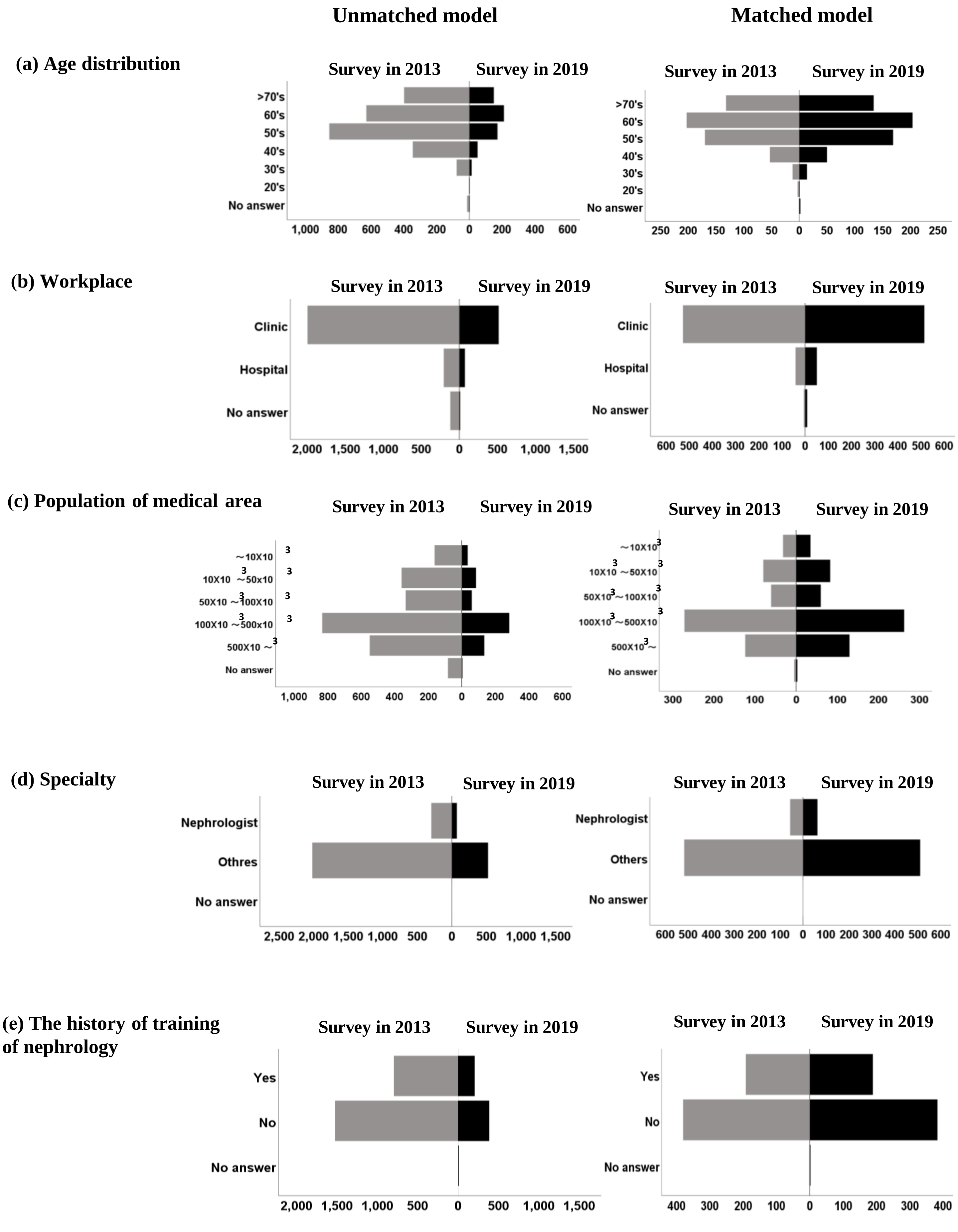

Supplement: Supplementary file 1 [file jcm-11-04779-s001.zip › jcm-1780788-supplementary.pdf]
